# Role of pore dilation in molecular transport through the nuclear pore complex: Insights from polymer scaling theory

**DOI:** 10.1371/journal.pcbi.1012909

**Published:** 2025-04-07

**Authors:** Atsushi Matsuda, Mohammad R K Mofrad

**Affiliations:** 1 Molecular Cell Biomechanics Laboratory, Departments of Bioengineering and Mechanical Engineering, University of California Berkeley, Berkeley, California, United States of America; 2 Molecular Biophysics and Integrative Bioimaging Division, Lawrence Berkeley National Laboratory, Berkeley, California, United States of America; Babes-Bolyai University: Universitatea Babes-Bolyai, ROMANIA

## Abstract

The nuclear pore complex (NPC), a channel within the nuclear envelope filled with intrinsically disordered proteins, regulates the transport of macromolecules between the nucleus and the cytoplasm. Recent studies have highlighted the NPC’s ability to adjust its diameter in response to the membrane tension, underscoring the importance of exploring how variations in pore size influence molecular transport through the NPC. In this study, we investigated the relationship between pore size and transport rate and proposed a mathematical model describing this connection. We began by theoretically analyzing how the pore size scales with the characteristic dimensions of the mesh-like structure within the pore. By introducing key assumptions about how the meshwork structure influences molecular diffusion, we derived a mathematical expression for the transport rate based on the size of the pore and the transported molecules. To validate our model, we conducted Brownian dynamics simulations using a coarse-grained representation of the NPC. These simulations, performed across a range of pore sizes, demonstrated strong agreement with our model’s predictions, confirming its accuracy and applicability. Our model is specifically tailored for small-to-medium-sized molecules, approximately 5 nanometers in size, making it relevant to a wide range of transcription factors and signaling molecules. It also extends to molecules with weak and transient interactions with FG-Nups, such as importin-*β*. By presenting this model formula, our study offers a quantitative framework for analyzing the effects of pore dilation on nucleocytoplasmic transport.

## Author summary

Recent research has suggested the role of the NPC dilation in enhancing molecular flux, noting that increased nuclear membrane tension can widen the NPC and promote the nuclear entry of certain transcription factors. Despite observations that NPC dilation can be experimentally induced, structural analyses reveal that NPC diameter variations are limited, typically ranging from 40 to 60 nm. This raises the question of whether such modest changes in pore size can effectively influence molecular flux. Our study addresses this by demonstrating an exponential relationship between pore size and transport rate, indicating that even minor adjustments in pore size can substantially increase molecular flux by several orders of magnitude. This result emphasizes the critical impact of pore size on molecular transport, highlighting the significance of controlling the NPC geometry for cellular functions.

## Introduction

Within the complex structure of eukaryotic cells, two distinct compartments are fundamental: the nucleus and the cytoplasm. Linking these two vital compartments is the nuclear pore complex (NPC), a protein assembly embedded in the nuclear envelope [1–6]. As the exclusive passageway between the nucleus and cytoplasm, the NPC plays a critical role in regulating molecular traffic. It adeptly facilitates the passage of essential molecules, including RNA and transcription factors, thereby enabling effective communication between the nucleus and cytoplasm [7,8]. Simultaneously, it acts as a barrier to prevent the entry of non-specific molecules, maintaining the integrity of the nuclear environment [9–11]. This selective permeability underscores the NPC’s vital role in cellular homeostasis and the precise control of genetic information flow.

**Fig 1:**
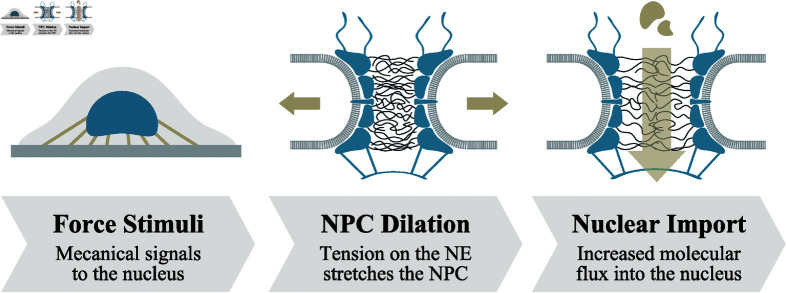
The influence of pore dilation on molecular transport. A force applied to the nuclear membrane induces the NPC to stretch, thereby enlarging the pore diameter and potentially leading to an increase in molecular flux.

Recent perspectives highlight the plasticity of the NPC and its role in diversifying the molecular flux through the pore (Fig 1) [12–14]. Various studies have demonstrated that the NPC can adjust its pore size in response to mechanical stimuli. For instance, Elosegui-Artola et al. [15,16] observed an increase in pore diameter and subsequent molecular influx into the nucleus when applying tension to the nuclear envelope. Conversely, Zimmerli et al. [17] reported NPC constriction under reduced tension on the nuclear envelope. The recent breakthroughs in defining the precise protein architecture of the NPC lend further support to its dynamic ability to adjust pore size through dilation and constriction [18–22]. These insights highlight the NPC’s structural flexibility, emphasizing its role in adapting selective transport in response to the cell’s mechanical environment.

While more evidence emerges about the structural flexibility of the NPC, our understanding of how pore dilation and constriction affect molecular flux remains limited. The relationship between pore size and molecular flux appears nonlinear and complex, as demonstrated by several experimental studies [23–25]. This complexity arises from the presence of intrinsically disordered proteins called FG-Nups on the inner surface of the NPC [26–30]. FG-Nups are characterized by their rich content of phenylalanine and glycine motifs, collectively known as FG-motifs, which engage in hydrophobic interactions amongst themselves. By occupying the NPC’s interior, FG-Nups effectively set a natural limit on the size of molecules that can pass through. This size limit changes dynamically depending on the global structure of the NPC and the density of FG-Nups. Adding further complexity, molecules capable of forming hydrophobic bonds with the FG-motifs can overcome this size restriction, enabling larger molecules to pass through the NPC [25,31].

To better understand the complex relationship between pore size and molecular transport rate, a quantitative model describing this relationship is essential. In this study, we proposed such a model by employing polymer scaling theory [32,33]. We demonstrated how variations in pore size scale with the characteristic length of the FG-Nup networks and analyzed their impact on molecular transport rates. The model was evaluated through Brownian dynamics simulations [34–37], in which dynamics of molecules and FG-Nups within the NPC were effectively replicated by solving Langevin equation. Our model effectively captured the transport behavior of relatively small molecules ( ∼ 5 nm in size), corresponding to transcription factors and signaling molecules.

The paper is organized as follows: In the Model section, we derive a model formula describing the relationship between NPC diameter and molecular transport rate. The Results section evaluates the accuracy of the model through Brownian dynamics simulations. In the Discussion section, we address the limitation of the model and its applicability in biological scenarios. Finally, the Methods section provides a detailed explanation of the simulation framework employed in this study.

## Model

In this section, we propose a model formula describing the relationship between the pore diameter, *D*, the cargo diameter, *d*, and the transport rate, $k_{AB}$. Our formulation starts by conceptualizing each FG-Nup as homopolymer following the scaling law, $R\approx bN^{\nu }$, where *R* is the end-to-end distance, *b* is the Kuhn length, *N* is the number of segments, and *ν* is the Flory exponent. The scaling relation enables us to effectively capture the statistical characteristics of polymer conformations, which has been extensively applied in studies of FG-Nups [38].

**Fig 2:**
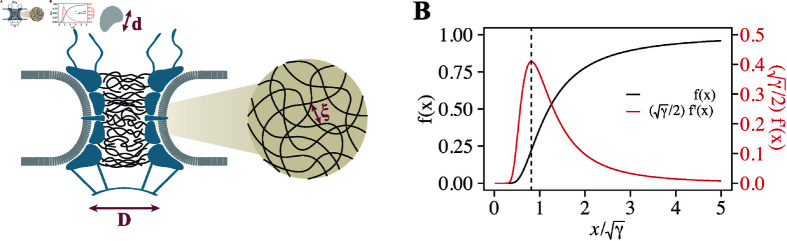
Theoretical model describing the relationship between pore size and transport rate. (A) Three critical length scales: pore diameter (*D*), cargo diameter (*d*), and mesh size (*ξ*). (B) Model function for the transport rate, *f*(*x*) (Eq. 6), and its derivative, $f^{\prime }(x)$. The parameter $x=\tilde{D}∕{\tilde{d}}^{\alpha ∕2}$ is the normalized pore size and *γ* is the dimensionless parameter. The derivative, $f^{\prime }(x)$, peaks at $x_{0}=\sqrt{2\gamma ∕3}$.

First, we explored how changes in pore size influence the characteristic length of the FG-Nup meshwork. We focused on the scenario where the polymer concentration within the NPC exceeds the overlap concentration, which is typically referred to as a semi-dilute polymer solution. The semi-dilute polymer solution has a characteristic length scale called correlation length, *ξ*. Conceptually, the correlation length is the average distance between distinct polymers within the solution (Fig 2A). Since the correlation length serves as a representative measure of the mesh-like structure of polymers, we refer to it as “mesh size”. On the basis of the scaling law, the mesh size can be quantified using the volume fraction of polymers, *ϕ*, as follows [32,33]:


$$\begin{array}{ccc} \xi \approx b\phi^{-\nu ∕(3\nu -1)}. & & \end{array}$$ (1)

We suppose that the volume fraction can be expressed as $\phi \approx AN∕D^{2}$, where *D* is the diameter of the pore, $A=4v_{mon}n_{FG}∕\pi h$ is the prefactor having a unit of squared area, $v_{mon}$ is the volume of the polymer segment, and *h* is the height of the pore.

When the mesh size is equal to the end-to-end distance, i.e. $\xi =\xi^{*}\approx bN^{\nu }$, the polymer solution is at overlap concentration. The volume fraction and the pore diameter at the overlap concentration becomes $\phi^{*}\approx N^{1-3\nu }$ and $D^{*}\approx A^{1∕2}N^{3\nu ∕2}$, respectively. With these scale parameters, we can derive the normalized form of Eq. 1 as:


$$\begin{array}{ccc} \xi ∕\xi^{*}\approx {\left(D∕D^{*}\right)}^{2\nu ∕(3\nu -1)}. & & \end{array}$$ (2)

Next, we estimated the free energy, *ΔG*, required to insert a molecule into the polymer solution. There are three length scales of interest in our set-up: the cargo’s diameter *d*, the pore’s diameter *D*, and the mesh size *ξ* (Fig 2A). When a molecule enters the polymer solution, it interacts with the local structure of the polymer, i.e. polymer mesh, instead of the entirety of the pore’s geometry. Therefore, we posit that the relevant length scales for the free energy calculation are solely the cargo size *d* and the mesh size *ξ*, resulting *ΔG* ∕ *kBT* ≈ *h* ( *d* ∕ *ξ* ) . Here, *kB* is Boltzmann constant, *T* is temperature, and *h* ( ⋅ )  is a dimensionless function. Additionally, we assume that the free energy is directly proportional to the polymer volume fraction, *ϕ* [39]. This is underpinned by the rationale that the number of monomers displaced by the molecule’s insertion scales with *ϕ*. Given that displacing each monomer requires a specific amount of work, the overall free energy scales with *ϕ*. By considering Eq. 1 and adjusting the exponent of the function *h* ( *d* ∕ *ξ* )  so that it scales with *ϕ*, we get


$$\begin{array}{ccc} \frac{\Delta G}{kBT}\approx \gamma {\left(\frac{d}{\xi }\right)}^{(3\nu -1)∕\nu }, & & \end{array}$$ (3)

where *γ* is a dimensionless constant. We can consider *γ* as a parameter that comprehensively describes the effect of various factors, such as the mutual polymer interactions or the detailed cargo geometry, which collectively contribute to the insertion barrier formation.

Finally, we formulated the transport rate of the molecule through the pore, $k_{AB}$. We assumed that the transport rate is directly related to the insertion free energy, *ΔG*. This is based on the assumption that the energy barrier for molecular insertion into the NPC is significantly larger than the energy associated with diffusion within the NPC, thereby dominating the overall transport process [25,40,41]. According to the Arrhenius formulation, we have


kAB=k0 exp ⁡  (−ΔGkBT), (4)

where $k_{0}$ is the transport rate of the molecule with an infinitesimally small diameter.

Combining Eqs. 2, 3, and 4, we get


$$\begin{array}{ccc} {\tilde{k}}_{AB}=\exp\left(-\gamma {\tilde{d}}^{\alpha }{\tilde{D}}^{-2}\right). & & \end{array}$$ (5)

Here, ${\tilde{k}}_{AB}=k_{AB}∕k_{0}$, is the normalized transport rate, $\tilde{d}=d∕\xi^{*}$, is the normalized cargo diameter, and $\tilde{D}=D∕D^{*}$ is normalized pore diameter. The exponent *α* = ( 3*ν* − 1 ) ∕ *ν* is a parameter that depends on the polymer’s scaling characteristics. By defining a parameter $x\equiv \tilde{D}∕{\tilde{d}}^{\alpha ∕2}$, which signifies the pore size normalized by the cargo size, the transport rate is rewritten such that


$$\begin{array}{ccc} {\tilde{k}}_{AB}\equiv f(x)=\exp(-\gamma ∕x^{2}). & & \end{array}$$ (6)

In Fig 2B, we illustrate this function (Eq. 6) and its derivative. While *f*(*x*) grows monotonically with *x*, its derivative $f^{\prime }(x)$ exhibits a distinct peak at $x_{0}=\sqrt{2\gamma ∕3}$. This suggests that for a given cargo size, the impact of pore dilation is most proounced at $x=x_{0}$. This is interpreted as follows: when the pore size is significantly small/large, it becomes consistently impermeable/permeable to the cargo, rendering the effect of pore dilation negligible. Conversely, when the pore size falls within a particular range yielding a mesh size comparable to the cargo size, any slight deviation in pore size effectively alters the cargo dynamics, resulting in the remarkable change in transport rate.

## Results

### Validation of the model formula through Brownian dynamics simulations

In this section, we present the results of the Brownian dynamics simulations [34–37], which were used to calculate the molecular transport rate through the NPC. These simulations aimed to evaluate the validity of our model formula (Eq. 6) describing the relationship between pore size and transport rate.

**Fig 3:**
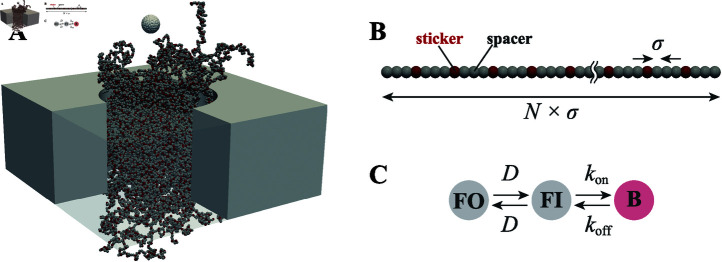
Coarse-grained simulation model of the NPC. (A) Representation of the coarse-grained model. The NPC is modeled as a cylinder with FG-Nups attaached to its inner surface. The cargo is modeled as a sphere. Both the FG-Nups and the cargo are represented by sets of discrete beads. (B) FG-Nup represented by the spacer-sticker model with a sticker placed every three spacers. (C) Transition dynamics of the sticker beads among three states: free outside (FO), free inside (FI), and bound (B). Transitions between FO and FI are dictated by diffusion, while transitions between FI and B follow a Monte Carlo approach, dictated by the binding and unbinding rates, $k_{on}$ and $k_{o\mathrm{ff}}$, respectively.

In the simulations, the NPC was represented as a coarse-grained particle-based system (Fig 3). The pore was modeled as a cylindrical conduit with diameter *D*, FG-Nups were depicted as bead-spring chains anchored to the pore surface, and the cargo, i.e. the molecule transported through the pore, was modeled as a spherical rigid body with diameter *d*. The system’s dynamics were computed by solving the overdamped Langevin equation [42,43] in conjunction with kinetic Monte Carlo methods [44]. Details of the simulation model are provided in the Methods section.

Before analyzing the molecular transport, we examined the structural properties of our coarse-grained system. As detailed in Appendix C in S1 Text, the system adhered to the scaling relation, a fundamental assumption of our theoretical framework. At the single-polymer level, the size of FG-Nups exhibited a power-law relationship with polymer length, such that $R∼N^{\nu }$. The Flory exponent was close to *ν* = 0 . 588, indicating that the simulation exists in a good solvent state rather than a condensed state. When FG-Nups are tethered to the NPC wall and form a polymer solution within the pore, a characteristic length corresponding to the mesh size, *ξ*, emerged. This length scaled with the pore diameter as $\xi ∕\xi^{*}∼{\left(D∕D^{*}\right)}^{2\nu ∕(3\nu -1)}$, lending support to our model formula (Eq. 2). These observations confirm that our simulation captures the structural features consistent with the assumptions of our model. Therefore, the simulation results presented below serve as an appropriate test case for validating our theoretical model.

In the following part of this section, we calculated the transport rate of the cargo through the NPC, $k_{AB}$. The transport rate was defined as the probability of the cargo successfully diffusing from one side to the other side of the pore within a given time. To reduce the computational cost to calculate the transport rate, we employed the forward flux sampling (FFS) method [45–47]. FFS is a computational technique to estimate the rate of stochastic transitions between two states, which, in this case, correspond to the cargo being on one side of the pore or the other. It works by dividing the transition pathway into a series of intermediate states, or “interfaces", and sequentially sampling trajectories that move forward from one interface to the next. This approach avoids the need to simulate the entire transition in a single step, significantly reducing computational expense while providing accurate rate calculations. The details of how this method was implemented in our system are provided in the Method section and Appendix B in S1 Text. Additionally, the accuracy of this method was validated against a control case, as detailed in Appendix B in S1 Text.

### Alignment of full and partial transport rates with the model formula

We first calculated the transport rate for a fixed length of FG-Nups, *N* = 209. This corresponds to the average length of FG-Nups observed in yeast NPC, which is 180 nm [26]. Using the FFS method, we calculated the transport rate for different pore diameters, *D* = 40, 50, and 60 nm, and analyzed the relationship between pore size and transport rate. Simulations were conducted for various cargo diameters, *d* = 1, 2, 3, 4, 5, and 6 nm. These cargoes were modeled as non-interactive with FG-Nups by treating all beads on the cargo surface as “spacers."

**Fig 4:**
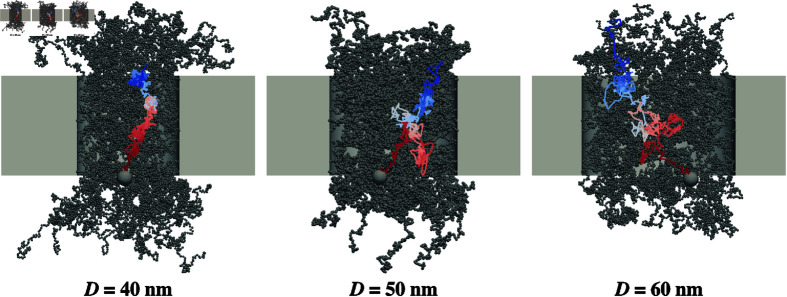
Trajectory of the cargo through the NPC. Trajectories of the cargo through pores of varying diameters (*D* = 40 , 50, and 60 nm) are visualized by a color gradient transitioning from blue to red. The cargo size is *d* = 6 nm and the length of FG-Nups is *N* = 209.

Snapshots of the FG-Nups and sample trajectories of the cargoes are shown in Fig 4. As the pore diameter increased, the density of FG-Nups within the pore decreased. This likely affected the diffusivity of the cargo within the pore, as observed in the larger fluctuations in the cargo’s trajectory with increasing pore size. Some trajectories exhibit the “backward" motion of the cargo (e.g., for *D* = 50 nm), where it temporarily moves against the direction of net transport. This behavior is consistent with our FFS framework, which allows for backward motion of the cargo with a specific probability. Trajectories that include backward motion but ultimately result in successful transport to the other side of the pore are assigned higher statistical weights, ensuring quantitative accuracy in the calculation of the overall transport rate (see Appendix B in S1 Text).

**Fig 5:**
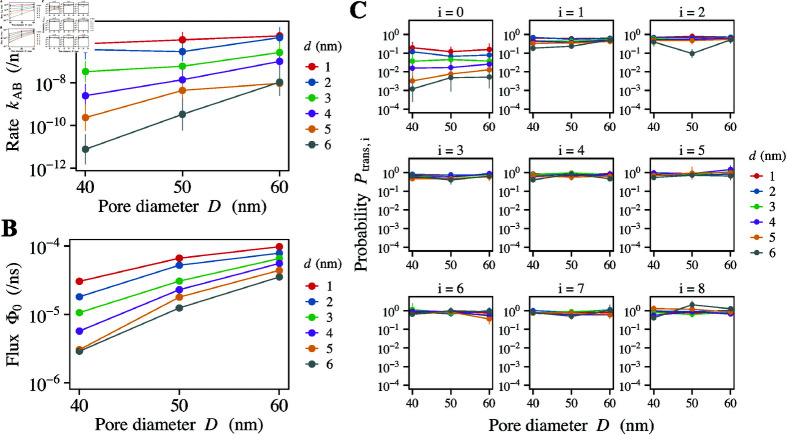
Transport rate calculated by FFS simulation. (A) Full transport rate, $k_{AB}$. (B) Flux into the first interface, $\Phi_{0}$. (C) Transition probability moving through each interface, $P_{trans,i}$ (*i* = 0 , 1 , . . . 8). The data is for polymer length *N* = 209. The horizontal axis shows the pore diameter, *D*, and the color legend represents the cargo diameter, *d*.

Our simulation results for the transport rate, $k_{AB}$, are presented in Fig 5A. As expected, increasing the pore size resulted in an enhancement of the transport rate across all cargo sizes. The transport rate can be decomposed into the flux to the first interface, $\Phi_{0}$, and the transition probabilities at each subsequent interface, $P_{trans,i}$ ($i=0,1,\ldots ,n_{FFS}$), such that $k_{AB}=\Phi_{0}\prod_{i=0}^{n_{FFS}}P_{trans,i}$, where $n_{FFS}=8$ represents the total number of interfaces. As shown in Fig 5B and C, the initial flux, $\Phi_{0}$, along with the transition probability at the 0-th interface, $P_{trans,0}$, are the principal determinants of the overall rate constant, while the transition probabilities at subsequent interfaces exert only a minor effect. This observation aligns with our theoretical model assumption, which posits that the insertion energy is the critical factor governing the overall transport process. As discussed in Appendix D in S1 Text, the partial transport rate, $k_{AB}^{\prime }=\Phi_{0}P_{trans,0}$, sufficiently captures the trend observed in the full transport rate, $k_{AB}$. In the subsequent parts of this study, we extensively use the partial transport rate, $k_{AB}^{\prime }$, for our analysis, given its equivalence to the full transport rate, $k_{AB}$.

**Fig 6:**
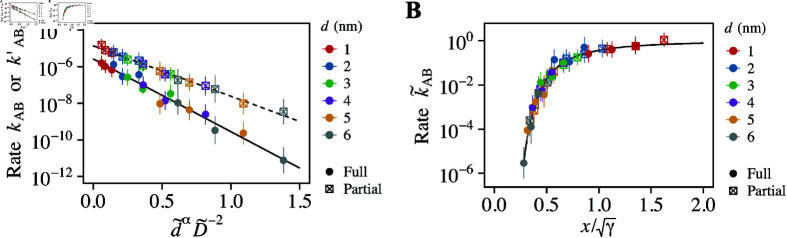
Validation of the model formula. (A) Normalized transport rate, ${\tilde{k}}_{AB}$, as a function of ${\tilde{d}}^{\alpha }{\tilde{D}}^{-2}$. Solid and dashed lines represent the linear regression of the data for full and partial transport rates, respectively. The slopes are denoted by *γ* = 9 . 15 for full transport and *γ* = 6 . 33 for partial transport. (B) Normalized transport rate, ${\tilde{k}}_{AB}$, as a function of $x=\tilde{D}∕{\tilde{d}}^{\alpha ∕2}$, adjusted by $\sqrt{\gamma }$. The solid line represents the model formula (Eq. 6).

We analyzed whether the simulation data adhered to our proposed model formula (Eq. 6). To do so, we first normalized the dimensions of cargo and pore diameter, $\tilde{d}=d∕\xi^{*}$ and $\tilde{D}=D∕D^{*}$, respectively, using following parameters: *N* = 209, *b* = *σ* = 0 . 86 nm, *ν* = 0 . 588, and $v_{mon}=\pi \sigma^{3}∕6=0.333$ nm^3^. We then plotted the full and partial transport rates, $k_{AB}$ and $k_{AB}^{\prime }$, as a function of ${\tilde{d}}^{\alpha }{\tilde{D}}^{-2}$ (Fig 6A). On the semi-logarithmic plot, the data aligned linearly, confirming the validity of the exponential relationship described in Eq. 5. By employing the linear regression, we determined the dimensionless constant, *γ*, and the baseline transport rate, $k_{0}$. For the full transport scenario, *γ* = 9 . 15 and $k_{0}=2.6\times 10^{-6}$ ns^-1^, while for the partial transport scenario, *γ* = 6 . 33 and $k_{0}=1.4\times 10^{-5}$ ns^-1^. The decrease in *γ* and the increase in $k_{0}$ from the full to the partial transport rate is reasonable, as the partial transport pathway is less restrictive, requiring fewer interfaces to be traversed compared to the full transport pathway.

Using the *γ* and $k_{0}$ values calculated above, we replotted the normalized transport rate, ${\tilde{k}}_{AB}=k_{AB}∕k_{0}$ or $k_{AB}^{\prime }∕k_{0}$, against the normalized pore diameter, $x=\tilde{D}∕{\tilde{d}}^{\alpha ∕2}$, scaled by the dimensionless constant $\sqrt{\gamma }$ (Fig 6B). The black line represents our model formula (Eq. 6), which aligned well with both full and partial transport data. These results confirms the validity of our theoretical model and demonstrate its comprehensive applicability to various scenarios when the data is appropriately scaled.

**Fig 7:**
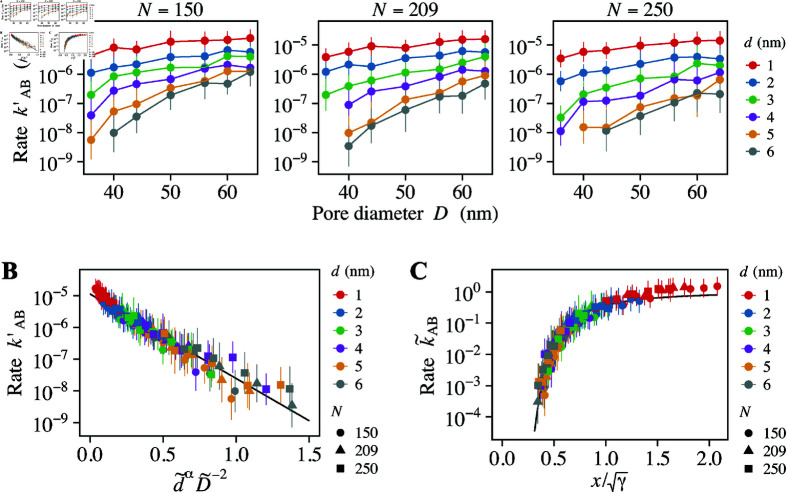
Effect of the polymer length. (A) Partial transport rate, $k_{AB}^{\prime }$, for different polymer length, *N* = 150, 209, and 250. (B) Normalized transport rate, ${\tilde{k}}_{AB}$, as a function of ${\tilde{d}}^{\alpha }{\tilde{D}}^{-2}$. The solid line represents the linear regression with the slope of *γ* = 6 . 13. (C) Normalized transport rate, ${\tilde{k}}_{AB}$, as a function of $x=\tilde{D}∕{\tilde{d}}^{\alpha ∕2}$, adjusted by $\sqrt{\gamma }$. The solid line represents the model formula (Eq. 6).

### Impact of FG-Nup length on mesh size and transport rate

To further validate our model formula, we simulated the transport dynamics with different length of FG-Nups, *N*. Changing the FG-Nup length alters the density of FG-Nups within the pore, thereby influencing the mesh size. Our model predicts that changes in mesh size significantly affect the transport rate. As such, examining whether the simulation data for different FG-Nup lengths align with our model formula provides a robust way to evaluate the validity of the model.

Guided by earlier findings that the initial flux effectively captures the overall transport behavior, we opted to compute the partial transport rate, $k_{AB}^{\prime }=\Phi_{0}P_{trans,0}$, rather than the full transport rate. We calculated the partial transport rate for various combinations of polymer length (*N* = 150, 209, 250), pore diameter (*D* = 36, 40, 44, 50, 56, 60, 64 nm), and cargo diameter (*d* = 1, 2, 3, 4, 5, 6 nm). For *N* = 150, 209, and 250, the pore diameters at the overlap concentration are calculated as $D^{*}=A^{1∕2}N^{3\nu ∕2}=76.4$, 102, and 120 nm, respectively. Since these values are all larger than the pore diameters tested in our simulations, the concentration of FG-Nups within the pore exceeds the overlap concentration. This condition satisfies a critical assumption of our theoretical model, thereby ensuring that these simulations are appropriate for validating the model.

The partial transport rates for each FG-Nup length *N* are presented in Fig 7A. Across all cases, the trend of higher transport rates for smaller cargoes and larger pores remained consistent. When comparing different polymer lengths, the transport rate decreased as the polymer length increased, which was especially prominent for larger cargoes and smaller pores. In some instances involving large cargoes and small pores, the transport rate dropped to zero; these data points were excluded from the plot for clarity.

We then investigated whether these data follows our model formula, Eq. 6. We proceeded to compute the normalized dimensions for pore and cargo, $\tilde{D}=D∕D^{*}$ and $\tilde{d}=d∕\xi^{*}$, respectively. Since the reference dimensions, given by $D^{*}=A^{1∕2}N^{3\nu ∕2}$ and $\xi^{*}=bN^{\nu }$, incorporate the polymer length, *N*, this normalization step ensures that the influence of polymer length is systematically incorporated into subsequent analyses. Fig 7B illustrates the linear relationship between the logarithm of the transport rate and the term ${\tilde{d}}^{\alpha }{\tilde{D}}^{-2}$, confirming the validity of our model. The data from all three cases fell on the same line. This suggests that varying the FG-Nup length does not affect the phenomenological parameters, *γ* and $k_{0}$. This observation is consistent with the origins of these parameters: *γ* is the coefficient that connects the insertion energy to the size ratio of the molecule and the mesh, while $k_{0}$ represents the base transport rate in the absence of insertion energy. Through linear regression analysis, we determined the values of the slope and intercept to be *γ* = 6 . 13 and $k_{0}=1.14\times 10^{-5}$ ${\text{ns}}^{-1}$, respectively. Using these values, we normalized the parameters and plotted normalized transport rate, ${\tilde{k}}_{AB}$, as a function of $x∕\sqrt{\gamma }$ (Fig 7C). The black line represents the model formula, Eq. 6. The close alignment of the simulation data with this formula provides further validation of our theoretical model across various FG-Nup lengths.

Although the simulation data generally conformed to the model formula, we observed a trend of upward deviation from the model as *x* grew larger and diverged further from $x_{0}=\sqrt{2\gamma ∕3}$ (Fig 7C). This is likely because, when *x* becomes too large, the cargo size is much smaller than the mesh size, rendering the FG-Nups barrier ineffective. Under these conditions, the free energy argument becomes inaccurate, revealing a limitation of our model. In Appendix D in S1 Text, we demonstrate that two mechanisms govern the transport rate: mesh-size-governed and pore-size-governed transport. When the cargo size is comparable to the mesh size, i.e., $x∼x_{0}$, the transport dynamics are well described by our theoretical model. However, when the mesh size significantly exceeds the cargo size, i.e., $x\gg x_{0}$, the FG-Nup mesh no longer acts as a barrier, and instead, the overall pore size determines the transport rate. This analysis underscores that our model formula, Eq. 6, is valid within a specific range of cargo sizes.

### Effect of attractive cargo surfaces on transport rate

Finally, we investigated the applicability of our model to cargoes that exhibit attractive interactions with FG-Nups. Up to this point, our analysis has focused on inert cargoes that interact with FG-Nups only through steric repulsion. However, in biological systems, cargoes such as importins and exportins possess hydrophobic surfaces that facilitate attractive interactions with FG-Nups. We investigated whether the transport of such attractive cargoes could be analyzed within the same framework as that of inert cargoes, as described by Eq. 6.

**Fig 8:**
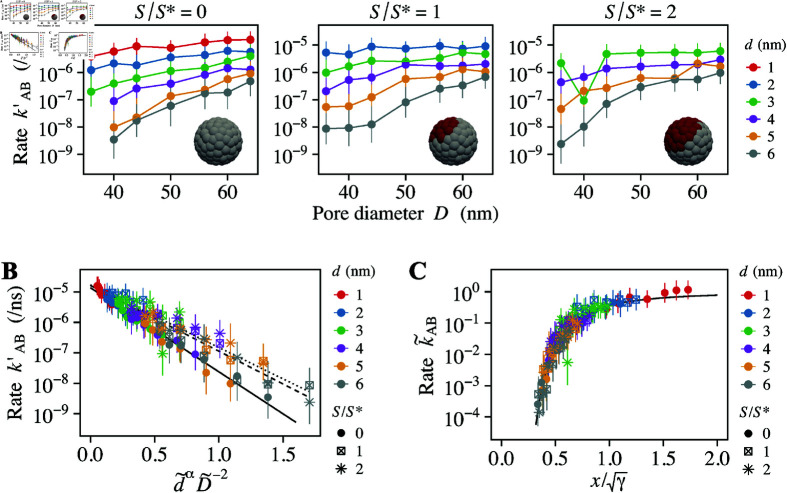
Effect of the attractive surface. (A) Partial transport rate, $k_{AB}^{\prime }$, for different attractive surface area, $S∕S^{*}=0$, 1, and 2. The reference surface area is $S^{*}=4\pi$ ${\text{nm}}^{2}$. The inset images depict cargo with a diameter *d* = 5 nm, featuring surfaces areas of attraction (red) and repulsion (gray). (B) Normalized transport rate, ${\tilde{k}}_{AB}$, as a function of ${\tilde{d}}^{\alpha }{\tilde{D}}^{-2}$. The solid, dashed, and dotted lines represent the linear regression of the data points for $S∕S^{*}=0$, 1, and 2, respectively. The slope of these lines are *γ* = 6 . 35 for $S∕S^{*}=0$, *γ* = 4 . 96 for $S∕S^{*}=1$, and *γ* = 4 . 73 for $S∕S^{*}=2$. (C) Normalized transport rate, ${\tilde{k}}_{AB}$, as a function of $x=\tilde{D}∕{\tilde{d}}^{\alpha ∕2}$, adjusted by $\sqrt{\gamma }$. The solid line represents the model formula (Eq. 6).

To model the attractive surface of the cargo, we designated a portion of the cargo beads as “stickers”. These stickers were designed to behave in exactly the same way as those of FG-Nups, i.e., interacting with stickers of FG-Nups via kinetic Monte Carlo scheme. We introduced a patch of attractive surface on the cargo, with an area of either $S∕S^{*}=1$ or 2, where $S^{*}=4\pi$ ${\text{nm}}^{2}$ is the reference surface area (see inset of Fig 8A). We conducted simulations and calculated the partial transport rate, $k_{AB}^{\prime }=\Phi_{0}P_{trans,0}$, using the following parameters: pore diameter (*D* = 36 , 40 , 44 , 50 , 56 , 60 , 64 nm), cargo diameter (*d* = 2 , 3 , 4 , 5 , 6 nm for $S∕S^{*}=1$ and *d* = 3 , 4 , 5 , 6 nm for $S∕S^{*}=2$), and FG-Nup length (*N* = 209).

The transport rates calculated from the simulations are shown in Fig 8A. For comparison, the data for inert cargo ($S∕S^{*}=0$) from the previous section is also included. The introduction of the attractive surface increased the transport rate. In Fig 8B, we plot the data against dimensionless parameters, ${\tilde{d}}^{\alpha }{\tilde{D}}^{-2}$. Cargoes with attractive surfaces, particularly those with $S∕S^{*}=2$, showed more variability than inert cargoes, but still they adhered to the predicted exponential relationship (Eq. 5). Through linear regression analysis, we identified the baseline transport rate, $k_{0}$, and the dimensionless parameter, *γ*. For inert cargoes, i.e. $S∕S^{*}=0$, the values were $k_{0}=1.36\times 10^{-5}$ ${\text{ns}}^{-1}$ and *γ* = 6 . 35. The addition of an attractive surface, with $S∕S^{*}=1$ and $S∕S^{*}=2$, led to increases in $k_{0}$ to $1.62\times 10^{-5}$ ${\text{ns}}^{-1}$ and $1.70\times 10^{-5}$ ${\text{ns}}^{-1}$, respectively, while *γ* decreased to 4.96 and 4.73. This quantitatively demonstrates that incorporating an attractive surface effectively elevated the transport rate. However, the changes in $k_{0}$ and *γ* were not directly proportional to the attractive surface area, $S∕S^{*}$. This suggests a nonlinear aspect of the phenomena and presents a complex challenge to quantitatively describe the transport dynamics of attractive cargoes. When displaying the normalized transport rate, ${\tilde{k}}_{AB}$, as a function of $x∕\sqrt{\gamma }$, where $x=\tilde{D}∕{\tilde{d}}^{\alpha ∕2}$ (Fig 8C), we observed that the data conformed to our model equation (Eq. 6), reinforcing the applicability of our model to scenarios involving attractive cargoes.

## Discussion

The molecular transport through the NPC is a fundamental mechanism that influences a broad spectrum of cellular processes. Recent research has highlighted the importance of pore dilation in modulating the flux of molecules into the nucleus. In our study, we introduced a theoretical model that delineates the relationship between the pore size and the transport rate and validated the model by using Brownian dynamics simulations. Our model formula provides a mathematical framework for quantitatively understanding and analyzing transport phenomena. In the following, we outline the conditions for applying this model, focusing on system dimensions and thermodynamic interactions, and discuss the biological scenarios where this model is applicable.

Firstly, our model requires a specific range of pore and cargo sizes relative to the FG-Nup concentrations. The model assumes that the pore is fully occupied by FG-Nups, with no empty regions. This condition is satisfied when $D<D^{*}$, where $D^{*}=A^{1∕2}N^{3\nu ∕2}$ represents the pore size at the overlap concentration of FG-Nups. For the yeast NPC, the average length of FG-Nups is 180 nm (*N* = 209) [26]. Using the parameters $v_{mon}=\pi \sigma^{3}∕6=0.333\;nm^{3}$, *h* = 40*nm*, $n_{FG}=80$, and *ν* = 0 . 588, the critical pore diameter is calculated as $D^{*}=102\;nm$. Given that structural studies estimate the pore size to range from *D* = 40 to 60*nm* [18,48–50], this condition is well satisfied.

As discussed in the Results section, the cargo size should be comparable to the mesh size. When the cargo size is too small, FG-Nups fail to act as an effective transport barrier. Conversely, when the cargo size is too large, transport requires substantial deformation of the FG-Nup structure, which falls beyond the scope of our model. Using the parameter values mentioned above, the mesh size is estimated to range from *ξ* = 4 . 6*nm* to 8 . 7*nm* for *D* = 40*nm* to 60*nm*. This range corresponds to the size of small to medium-sized biomolecules, such as importin-*β* ( ∼ 5*nm* [51]) or RanGTP ( ∼  4-5 nm [52]). Many transcription factors falls within this range, such as NF-*κ*B ( ∼  5-7 nm [53]), p53 ( ∼  5-6 nm [54]), or YAP ( ∼ 6*nm* [55]). It also includes short nucleic acids with secondary structures and small ribonucleoprotein complexes, such as snRNPs [56]. Viral capsids, such as those of the human immunodeficiency virus ( ∼  100–120 nm [57]) or hepatitis B virus ( ∼  30–36 nm [58]), are significantly larger than the mesh size, and therefore fall outside the scope of our model. For investigations on the transport of such large cargoes, readers may refer to other studies [59–61].

Secondly, our model is limited to cases where the attractions between FG-Nups are sufficiently weak. The model assumes that FG-Nups exist in a good solvent state, meaning that the overall repulsions between polymer segments dominate over attractions. When this condition is not met and attractions become dominant, FG-Nups collapse into a condensed state, effectively blocking the diffusion-mediated passage of cargo through the pore. Several metrics can assess this condition, as outlined in our review paper [38]. One example is the Flory exponent, *ν*, which characterizes how the radius of gyration scales with polymer length: *ν* < 1 ∕ 2 indicates a poor solvent (collapsed state), while *ν* > 1 ∕ 2 represents a good solvent (extended state). The solvent state of FG-Nups can vary between collapsed and extended, depending on the specific type of FG-Nup and the surrounding environment. For instance, Nup98 is collapsed in bulk solution but becomes extended within the NPC environment [62]. We hypothesize that the coexistence of various types of FG-Nups within the pore reduces the overall cohesiveness, maintaining a solvent state that approximates a good solvent. However, this interpretation remains a topic of active discussion and investigation.

The attractions between the cargo and FG-Nups must also be weak, as our model does not explicitly incorporate these interactions into the free energy calculation. When the attraction becomes too strong, the diffusive motion of the cargo is suppressed, potentially altering the transport rate. Interestingly, our simulation results for cargoes with attractive surfaces aligned with the predictions of our model formula, with the effects of attraction captured within the phenomenological parameter, *γ*. This agreement may stem from our specific approach to modeling attractive interactions using the kinetic Monte Carlo method, which effectively ensures that the interactions remain weak and transient. These findings suggest that, as long as the cargo-FG-Nup attractions are sufficiently weak, our model formula remains valid. As a next step, it will be important to investigate the range of attraction strengths within which our model formula is applicable. Experimentally, the attraction between cargoes and FG-Nups is commonly quantified using the dissociation constant, $K_{D}$. For example, the dissociation constant between Nup153 and importin-*β* was measured to be 1-8 mM [63]. In our simulation system, the dissociation constant is approximately $K_{D}=10\;mM$, based on the assumed diffusion-limited on-rate of $k_{on}=10^{9}\;M^{-1}s^{-1}$ [64] and our manually defined off-rate of $k_{o\mathrm{ff}}=2\times 10^{-4}\;\Delta t^{-1}$. This comparison suggests that the interaction dynamics of importin-*β* are similar to those in our model system and that importin-*β* is likely to follow the predictions of our model formula.

Lastly, we compare the model derived in this paper with other models proposed previously. To our knowledge, the study by Klughammer et al. [23] is the only one investigating a similar aspect. They conducted both simulations and experiments to propose that the transport rate changes quadratically with pore diameter, expressed as $k_{AB}∼{\left(D\;-\;D_{0}\;-\;d\right)}^{2}$, where $D_{0}$ is a shifting constant. The rationale behind their formula is that the transport rate increases proportionally with the area of the “hole," where FG-Nups are absent within the pore. This formulation implicitly considers scenarios where the pore diameter, *D*, exceeds the critical diameter for overlapping concentration, $D^{*}$. In contrast, our study focuses on cases where the pore diameter, *D*, is smaller than the critical diameter, $D^{*}$, where pore space is fully filled with FG-Nups. Thus, the two models are complementary, as they address distinct regimes of pore diameter relative to $D^{*}$, offering a comprehensive understanding of the transport phenomena.

In summary, this study proposed a model formula to describe the relationship between transport rate and pore diameter. Our model suggests that below the critical diameter, pore dilation can exponentially enhance molecular flux through the NPC. Given that the NPC diameter is generally within the range of 40-60 nm, which remains below the critical diameter discussed above, our findings provide valuable insights into the role of pore dilation in biologically relevant contexts. Specifically, this work supports the plausibility of the recent hypothesis linking the NPC to mechanotransduction [12,13,15,16], as molecules involved in this process, such as YAP, fall within the size range addressed by our model. While our model is examined through simulations, future experimental validation will be crucial to further assess its applicability and identify potential limitations. By presenting a mathematical formula linking pore dimensions to transport rates, this work contributes to a deeper understanding of NPC transport dynamics.

## Methods

### Coarse-grained model

The simulations were conducted in a $L_{x}\;\times \;L_{y}\;\times \;L_{z}$ simulation box with periodic boundary conditions applied on each face. To model the nuclear envelope (NE), a partition of thickness *h* was placed at the half plane of the simulation box. A cylindrical hole of diameter *D* was placed in the center of the NE to represent the NPC (Fig 3A). The NE and inner wall of the NPC were treated as hard surfaces.

We modeled the inner wall of the NPC by attaching $n_{FG}$ pieces of FG-Nups, each of which was represented as a series of bead-springs containing *N* beads, with each bead having a diameter of *σ* (Fig 3B). The beads were classified into two types: stickers, representing FG-motifs, and spacers, representing other residues [65]. To reflect the ratio of FG-motifs in FG-Nups of the yeast NPC, we placed one sticker in every three spacers [25,26]. To model the transported molecules, we used a spherical cargo of diameter *d*, whose surface was discretized into a set of beads of the diameter *σ*. The cargo surface beads were classified as stickers or spacers based on whether they had an attraction with FG-repeats or not.

In the following modeling section, we express the unit of variables using the bead diameter *σ* = 0 . 86 (nm), integration time step *Δt* = 0 . 02 (ns), and the thermal energy $kBT=4.28\times 10^{-18}$ (${\text{nm}}^{2}\cdot \text{g}\cdot {\text{ns}}^{-2}$), where *kB* is Boltzmann constant, *T* is temperature. All parameters used in this study are summarized in Tables 1 and 2.

**Table 1:** Parameters employed in our simulations. The values listed here were consistently used throughout our calculations.

Symbol	Value	Unit	Description
$L_{x}$	100	nm	X-length of the simulation box
$L_{y}$	100	nm	Y-length of the simulation box
$L_{z}$	200	nm	Z-length of the simulation box
*h*	40	nm	Thickness of the NE
$n_{FG}$	80	-	Number of FG-Nups
*σ*	0.86	nm	Diameter of the bead
*Δt*	0.02	ns	Integration time step
*kBT*	4.28$\times 10^{-18}$	${\text{nm}}^{2}\cdot \text{g}\cdot {\text{ns}}^{-2}$	Thermal energy at *T* = 310.15 (K)

**Table 2:** Parameters employed in our simulations. The values listed here were consistently used throughout our calculations.

Symbol	Value	Unit	Description
*η*	37.1	$kBT\cdot \Delta t\cdot \sigma^{-3}$	Viscosity of the medium (*η* = 5 . 0 cP)
$k_{b}$	100	$kBT\cdot \sigma^{-2}$	Spring constant of FG-Nups
$r_{eq}$	1	*σ*	Equilibrium distance between beads
$k_{a}$	0.5	*kBT*	Bending rigidity of FG-Nups
$\theta_{eq}$	*π*	rad	Equilibrium angle
$\epsilon_{r}$	400	*kBT*	Repulsion energy
$\sigma_{r}$	1	*σ*	Characteristic length for repulsion
$r_{cri}$	1	*σ*	Cutoff distance for repulsion
$F_{wall}$	400	$kBT\cdot \sigma^{-1}$	Constant force for hard wall repulsion
$r_{thre}$	2	*σ*	Threshold distance for FO ↔ FI transition
$F_{FG}$	2	$kBT\cdot \sigma^{-1}$	Constant force between bound (B) stickers
$k_{on}$	$2.0\times 10^{-3}$	${\Delta t}^{-1}$	Rate constant for IF → B transition
$k_{o\mathrm{ff}}$	$2.0\times 10^{-4}$	${\Delta t}^{-1}$	Rate constant for B → IF transition

### Brownian dynamics

We simulated the dynamics of the beads constituting FG-Nups by the overdammped Langevin equation [42,43],


$$\begin{array}{ccc} \zeta \frac{dr_{i}}{dt}=-\frac{\partial U}{\partial r_{i}}+\Lambda_{i}, & & \end{array}$$ (7)

where $r_{i}$ is the position of bead *i*, *ζ* is the friction coefficient, *U* is the potential energy function, and $\Lambda_{i}$ is the random force on bead *i*. According to Stokes’ law, we used *ζ* = 3*πησ*, where *η* is the viscosity of the medium. The random force satisfies,


$$\begin{array}{ccc} \left\langle \Lambda_{i\alpha }(t)\right\rangle =0 & & \end{array}$$ (8)

$$\begin{array}{ccc} \left\langle \Lambda_{i\alpha }(t)\Lambda_{j\beta }(t^{\prime })\right\rangle =2\zeta kBT\delta (t-t^{\prime })\delta_{ij}\delta_{\alpha \beta }, & & \end{array}$$ (9)

where the bracket indicates the ensemble average, $\delta (t-t^{\prime })$ is Dirac delta function, and $\delta_{ij}$ and $\delta_{\alpha \beta }$ are Kronecker’s delta. Subscripts *α* and *β* indicate *α*- and *β*- component of the vector. The potential energy, *U*, is written as,


$$\begin{array}{ccc} U=\underset{\left\langle i,j\right\rangle }{\sum }U_{i,j}^{bond}+\underset{\left\langle i,j,k\right\rangle }{\sum }U_{i,j,k}^{angle}+\underset{i,j}{\sum }U_{i,j}^{steric}, & & \end{array}$$ (10)

where $U_{i,j}^{bond}$ is the elastic energy of FG-Nups, $U_{i,j,k}^{angle}$ is the bending energy of FG-Nups, and $U_{i,j}^{steric}$ is the steric repulsion. The summations for the first and second terms are run over the neighboring beads pair within FG-Nups, while the summation for the third term are run over all beads pair in the system. Details of each potential is shown below:


$$\begin{array}{ccc} U_{i,j}^{bond} & =k_{b}{\left(r_{ij}-r_{eq}\right)}^{2} & \end{array}$$ (11)

$$\begin{array}{ccc} U_{i,j,k}^{angle} & =k_{a}\left\{1-\cos(\theta_{ijk}-\theta_{eq})\right\} & \end{array}$$ (12)

$$\begin{array}{ccc} U_{i,j}^{steric} & =\{\begin{array}{cc} U^{rep}(r_{ij}),\; & \text{for }r_{ij}<r_{cri}\\ U^{rep}(r_{cri}),\; & \text{otherwise} \end{array} & \end{array}$$ (13)

Urep(r)=ϵr exp ⁡ (−r∕σr) (14)

where $k_{b}$ is the spring constant, $r_{ij}$ is the distance between bead *i* and *j*, $r_{eq}$ is the equilibrium distance between two neighboring beads, $k_{a}$ is the bending rigidity, $\theta_{ijk}$ is the angle formed between bead *i*, *j*, and *k*, $\theta_{eq}$ is the equilibrium angle, $\epsilon_{r}$ is the repulsion energy, $\sigma_{r}$ is the characteristic length for repulsion, and $r_{cri}$ is the cutoff distance. The over-damped Langevin equation (Eq. 7) was integrated using a finite time step *Δt*.

When the distance between the bead’s center and the hard surface, i.e. the NE and the inner wall of the NPC, becomes less than *σ* ∕ 2, a constant force $F_{wall}$ is exerted on the bead. This force acts in a direction normal to and opposite from the surface.

The cargo was modeled as a rigid body, endowed with both translational and rotational degrees of freedom. Its dynamics are characterized by the translational and rotational Brownian motion. Interactions between the cargo and the FG-Nups are mediated through steric repulsion and sticker-sticker attraction. A comprehensive description of the modeling approach and the algorithm used to simulate the dynamics of the cargo can be found in the Appendix A in S1 Text.

### Kinetic Monte Carlo

In parallel with solving the Langevin equation, we employed the kinetic Monte Carlo method to simulate the dynamic interactions between stickers [44]. Each sticker was categorized into one of three states: free outside (FO), free inside (FI), or bound (B) (Fig 3C). A bead (FO) was defined as a sticker with no other stickers within a distance of $r_{thre}$ from its center, while a bead (FI) was defined as a sticker with more than one stickers within that distance. The FO  ↔  FI transition occurred via the diffusion described by Eq. 7. The FI  ↔  B transition occurred in a Monte Carlo fashion, with on and off rates of $k_{on}$ and $k_{o\mathrm{ff}}$, respectively. Once a sticker became bound (B), a constant force $F_{FG}$ towards the other attracted bead was applied. We imposed a restriction that each sticker could only bind to one other sticker and that FO  ↔  B transitions were forbidden.

### Forward flux sampling

To determine the rate at which cargo is transported through the NPC, we utilized the forward flux sampling (FFS) technique [45–47]. FFS belongs to the family of transition path sampling methods, designed specifically to compute the rate constants for barrier-overcoming events. This approach involves defining an order parameter that increases as the system progresses across the barrier. Interfaces are then established at specific intervals of the order parameter’s value. The simulation’s objective is to ascertain the likelihood of the system crossing each interface. The overall rate constant is derived from multiplying the flux into the first interface by the probabilities of crossing subsequent interfaces.

The comprehensive description of the algorithm and implementation of FFS in our simulation can be found in Appendix B in S1 Text. The following paragraphs provide an overview of the procedure.

In our analysis, the cargo’s center position was selected as the order parameter. We divided the simulation space into several distinct regions. The initial interface, labeled $I_{0}$, was positioned as a hemispherical region with a radius of *D* ∕ 2, centered at the NPC’s entrance. This demarcated the first basin, $B_{A}$, as the area outside this hemisphere. The central region of the pore was further segmented horizontally into $n_{FFS}$ cylindrical segments of equal size, each with a radius of *D* ∕ 2 and a height of $h∕n_{FFS}$. These segments, arranged vertically through the pore, defined the interfaces from $I_{1}$ to $I_{n_{FFS}}$. The space below the lowest interface, beyond the central pore region, was designated as the second basin, $B_{B}$.

The initial step involved calculating the flux, $\Phi_{0}$, representing the rate at which the system transitions from the first basin $B_{A}$ to the 0-th interface $I_{0}$. This flux was quantified by dividing the number of transitions into $I_{0}$ by the total simulation time of the flux calculation. Subsequently, we determined the probability of the system crossing each interface $I_{i}$, denoted as $P_{trans,i}$ ($i=1,2,...,n_{FFS}$). The methodology for calculating both the flux $\Phi_{0}$ and the transition probabilities $P_{trans,i}$ is detailed in the Appendix B in S1 Text. The transport rate, $k_{AB}$, representing the rate constant at which cargo passes through the NPC, was calculated using the formula:


$$\begin{array}{ccc} k_{AB}=\Phi_{0}\overset{n_{FFS}}{\underset{i=0}{\prod }}P_{trans,i}. & & \end{array}$$ (15)

## Supporting information

S1 Text. Supplemental material. (PDF)
